# Nrf2 in cancers: A double‐edged sword

**DOI:** 10.1002/cam4.2101

**Published:** 2019-03-30

**Authors:** Shijia Wu, Hong Lu, Yongheng Bai

**Affiliations:** ^1^ Key Laboratory of Diagnosis and Treatment of Severe Hepato‐Pancreatic Diseases of Zhejiang Province The First Affiliated Hospital of Wenzhou Medical University Wenzhou China; ^2^ Department of Laboratory Medicine The First Affiliated Hospital of Wenzhou Medical University Wenzhou China

**Keywords:** cancer, chemoresistance, inflammation, Keap1, Nrf2

## Abstract

The Nrf2/Keap1 pathway is an important signaling cascade responsible for the resistance of oxidative damage induced by exogenous chemicals. It maintains the redox homeostasis, exerts anti‐inflammation and anticancer activity by regulating its multiple downstream cytoprotective genes, thereby plays a vital role in cell survival. Interestingly, in recent years, accumulating evidence suggests that Nrf2 has a contradictory role in cancers. Aberrant activation of Nrf2 is associated with poor prognosis. The constitutive activation of Nrf2 in various cancers induces pro‐survival genes and promotes cancer cell proliferation by metabolic reprogramming, repression of cancer cell apoptosis, and enhancement of self‐renewal capacity of cancer stem cells. More importantly, Nrf2 is proved to contribute to the chemoresistance and radioresistance of cancer cells as well as inflammation‐induced carcinogenesis. A number of Nrf2 inhibitors discovered for cancer treatment were reviewed in this report. These provide a new strategy that targeting Nrf2 could be a promising therapeutic approach against cancer. This review aims to summarize the dual effects of Nrf2 in cancer, revealing its function both in cancer prevention and inhibition, to further discover novel anticancer treatment.

## 1 INTRODUCTION

Nuclear factor (erythroid‐derived 2)‐like 2 (Nrf2) encoded by *NFE2L2* gene belongs to a transcription factor subfamily with a Cap “n” Collar (CNC) structure and contains a basic leucine zipper DNA binding domain (b‐Zip) at the C terminus. Nrf2 possesses six highly conserved domains named Nrf2‐ECH homology (Neh) domains. The bZip in Neh1 domain allows Nrf2 to heterodimerize with small musculoaponeurotic fibrosarcoma proteins (sMafs).^1^ The Neh2 domain allows Nrf2 to bind and regulate its cytoplasmic chaperone molecule Kelch‐like‐ECH‐associated protein 1 (Keap1) in physiological manner.^2^ The two motifs of Neh2 domain, ETGE and DLG, bind to similar sites on the bottom surface of the Keap1 Kelch motif.^3^ Neh3 domain is necessary for protein stability and transcriptional activation.^4^ Neh4 and Neh5 function as two transactivation domains by interacting with CREB‐binding protein (CBP).^2^ Neh6 domain is rich in serine residues and contains a degron that is involved in the degradation of Nrf2 in oxidatively stressed cells.^5^ The activity of this degron could be increased by glycogen synthase kinase‐3 (GSK‐3) activity, suggesting that the stimulation of the degron of Neh6 domain could be an effective method to overcome the constitutive upregulation of Nrf2.^6^

## 2 NRf2 ACTIVATION

### 2.1 Keap1/Nrf2/ARE signaling pathway

**Figure 1:**
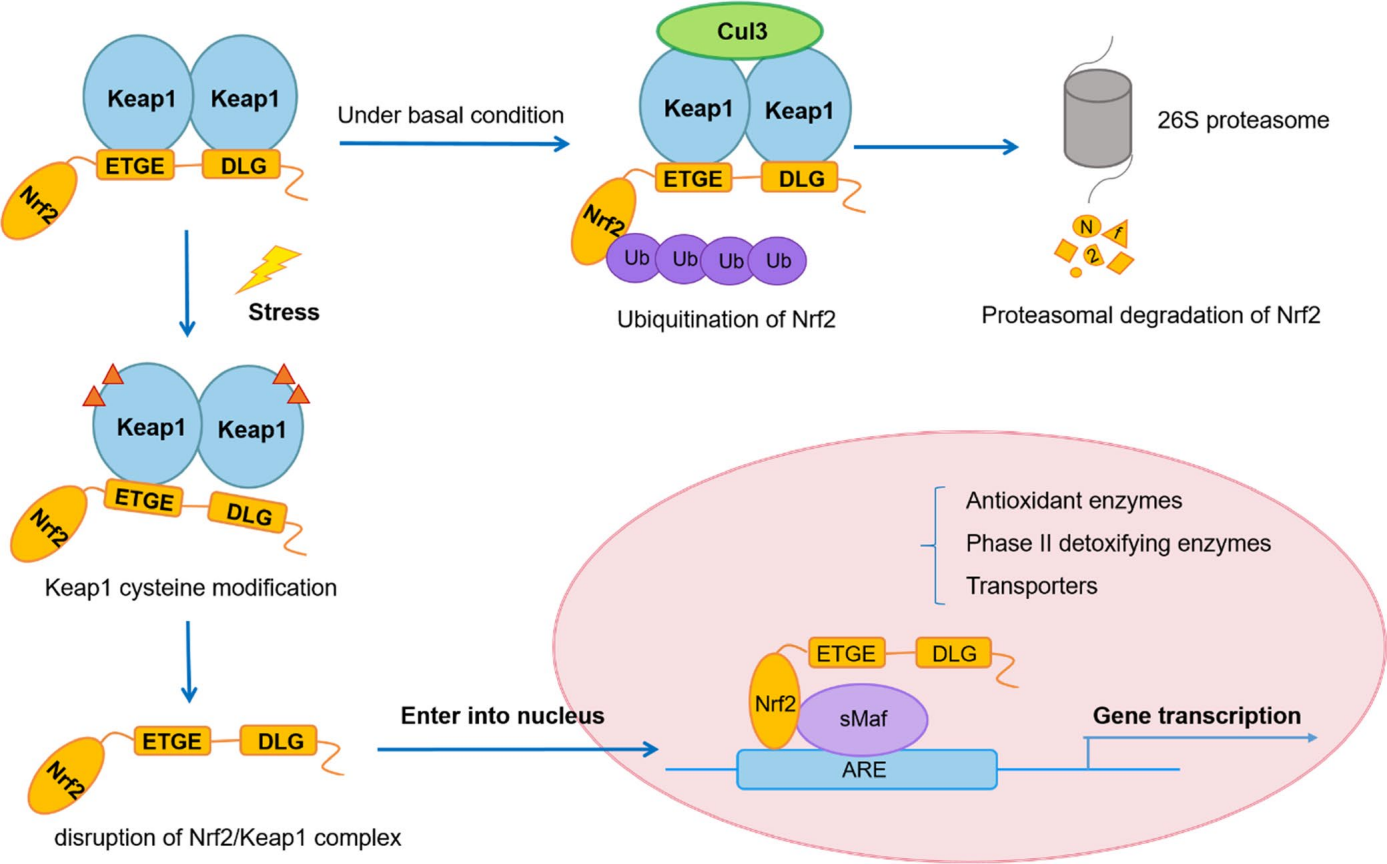
Nrf2/Keap1 signaling pathway. Under basal conditions, Nrf2 binds to Keap1 by its two motifs (ETGE and DLG) and activates Cul3‐mediated ubiquitination followed by proteasomal degradation. Under stress conditions, due to the modification of Keap1 cysteine residues, Nrf2 dissociates from Keap1 and translocates into the nucleus. Nrf2 then forms a heterodimer with sMaf protein and binds to ARE to initiate the transcription of various downstream genes

The Keap1‐Nrf2 system is of primary importance in maintaining cellular homeostasis in order to respond adaptively to xenobiotic and oxidative stress. Under basal condition, Nrf2 interacts with two molecules of Keap1 through its Neh2 ETGE and DLG motifs to activate Cullin 3 (Cul3)‐based E3 ligase complex‐mediated Nrf2 ubiquitination reaction.^7^ Once Nrf2 is ubiquitinated, it will be rapidly degraded by 26S proteasome and maintained at a very low level in the cytoplasm.^8^ On exposure of cells to oxidative stress or chemopreventive compounds, the cysteine residues of Keap1 are modified and the conformation changes, resulting in the detachment of Nrf2 DLG motifs from Keap1, disrupting the ubiquitination and degradation of Nrf2. The binding affinity between Nrf2 and Keap1 is reduced and the ubiquitination system of Nrf2‐Cul3 is disrupted. This allows the de novo‐synthesized Nrf2 to translocate into nucleus, forms a heterodimer with one of sMafs, and binds to antioxidant element (ARE) in the upstream promoter region of multiple genes. On recovery of the redox balance, Nrf2 is dissociated from the ARE sequence. Keap1 enters into the nucleus and escorts Nrf2 out of the nucleus to the cytoplasmic Cul3‐E3 ubiquitin ligase machinery for degradation. Ultimately, a low level of Nrf2 is reattained; the Nrf2/Keap1 signaling pathway is switched off (Figure 1).^9^, ^10^

### 2.2 Downstream targets of Nrf2

The Nrf2 downstream targets are classified into three major groups: phase I and phase II drug metabolizing enzymes as well as phase III drug transporters (Table 1). Briefly, phase I enzymes oxidize drugs or xenobiotics; while phase II enzymes conjugate products of phase I reactions, phase III enzymes transport the final metabolites out of cells, cooperating to exert a cytoprotective function. Phase I‐metabolizing enzymes oxidize, reduce, or hydrolyze xenobiotics and drugs, such as aldo‐keto reductases (AKR) and cytochrome P450s (CYPs) encoded by genes regulated by Nrf2.

**Table 1:** Downstream targets regulated by Nrf2

Abbreviation	Name	General biochemical function	Ref.
Xenobiotic detoxification			
NQO1	NAD(P)H:quinone oxidoreductase 1	Degradation of reactive quinone and scavenge of superoxide	11, 12
AKR	Aldo‐keto reductases	Reduce aldehydes and ketones	13
CBR	carbonyl reductase	prostaglandin metabolism	14
ADH	Alcohol dehydrogenase	Alcohol metabolism	15
ALDH	Aldehyde dehydrogenase	Catalyzes the oxidation of aldehydes	16
CYPs	cytochrome P450s	Catalyze the oxidation, reduction, and dehalogenation of various xenobiotics	17
CES	Carboxylesterases	Catalyze the hydrolysis of ester‐ and amide‐containing chemicals	18
SOD	Superoxide dismutase	Catalyzes the dismutation of the superoxide	19
EPHX1	Epoxide hydrolase	Catalyzes the hydrolysis of arene and aliphatic epoxides	20
Conjugation			
UGT	UDP‐glucuronosyltransferase	Conjugates glycosyl group with xenobiotics for detoxification	21
GST	Glutathione S‐transferases	Conjugate reduced GSH with xenobiotics for detoxification	22
SULT	sulfotransferases	Conjugate sulfate group with xenobiotics for detoxification	23
NAT	N‐acetyltransferase	Conjugates acetyl group with xenobiotics for detoxification	24
GSH metabolism			
xCT	the subunit of system xc–	Imports cysteine into the cell	25
GCLC	Glutamate‐cysteine ligase	Catalytic subunit in rate‐limiting step of GSH synthesis	26
GCLM	Glutamate‐cysteine ligase	Modifier subunit in rate‐limiting step of GSH synthesis	26
GPX	Glutathione peroxidase	Catalyzes the oxidation of GSH	27
GSR	Glutathione reductase	Catalyzes the NADPH‐dependent reduction of GSSG	22
Thioredoxin enzyme system			
Prxs	Peroxiredoxins	Catalyze the reduction of peroxides	28
Txn1	Thioredoxin‐1	Catalyzes the reduction of oxidized proteins	29
TrxR1	Thioredoxin reductases‐1	Catalyze the NADPH‐dependent reduction of oxidized Trx	30
Srxn1	Sulfiredoxin‐1	Reactivates Prxs	31
Heme metabolism			
HO‐1 (HMOX1)	Heme oxygenase 1	Cleaves heme to form biliverdin for the degradation of heme	32
BLVR	Biliverdin reductase	Reduction of biliverdin to bilirubin for the degradation of heme	33
FECH	Ferrochelatase	Converts Fe2+ to heme for the degradation of heme	34
FTH	Ferritin heavy chain	Storage of Fe for degradation of heme	35
FTL	Ferritin light chain	Storage of Fe for degradation of heme	35
NADPH generation and PPP pathway			
G6PD	Glucose‐6‐phosphate 1‐dehydrogenase	NADPH production in oxidative phase of PPP	36
PGD	6‐phosphogluconate dehydrogenase	NADPH production in oxidative phase of PPP	36
IDH1	Isocitrate dehydrogenase 1	NADPH production in oxidative phase of PPP	36
ME1	Malic enzyme 1	NADPH production, pyruvate regeneration for TCA cycle	36
TKT	Transketolase	directing carbon flux in nonoxidative phase of PPP	36
TALDO1	Transaldolase1	directing carbon flux in nonoxidative phase of PPP	36
Xenobiotic transporters			
MRPs	Multidrug resistance‐associated proteins	Transport or excrete drug metabolites out of cells	37
OATP2	Organic anion‐transporting polypeptide	Mediates the Na+‐independent uptake of organic anions	38
P‐gp	P‐glycoprotein	an ATP‐dependent efflux pump of wide range of xenobiotics	39
Fatty acid synthesis and oxidation			
ACL	ATP‐citrate lyase	Synthesis of cytosolic acetyl‐CoA for fatty acid synthesis	40
ACC	Acetyl‐CoA carboxylase	Synthesis of malonyl‐CoA for fatty acid synthesis	41
FASN	Fatty acid synthase	Synthesis of long‐chain saturated fatty acid for fatty acid synthesis	40
SCD	Stearoyl CoA desaturase	Introduction of a double bond for unsaturated fatty acid synthesis	40
Purine biosynthesis			
PPAT	Phosphoribosyl pyrophosphate amidotransferase	Catalyzes the rate‐limiting step in the de novo purine biosynthetic pathway	36
MTHFD2	Methylenetetrahydrofolate dehydrogenase 2	Provides one‐carbon units for purine biosynthesis	36
Transcription factors			
AhR	Aromatic hydrocarbon receptor	Promotes the expression of cytochrome P450s (CYPs) for inhibition of adipogenesis	42
PPARγ	Peroxisome proliferators activator receptors gamma	Promotes the expression of CYP4A gene for adipocyte differentiation and reduction in inflammation	43
CEBPα	CCAAT/enhancer‐binding protein alpha	Binds to the CCAAT box motif in various gene for adipocyte differentiation and macrophage function	44
RXRα	Retinoid X receptor alpha	Interacts with Neh7 domain of Nrf2 for the inhibition of Nrf‐Keap1 pathway	45

Phase II drug‐metabolizing enzymes regulated by Nrf2 are mostly engaged in metabolic pathways through metabolizing xenobiotics via glucuronidation, glutathione conjugation, and sulfation. The primary function of intracellular redox‐balancing proteins is to maintain the cellular levels of glutathione (GSH) and thioredoxin (Trx), which scavenge reactive oxygen species (ROS) and reactive nitrogen species (RNS) in cells.^46^ Nrf2/Keap1 signaling governs the expression of the xCT (aka SLC7a11 or system xc^–^),^25^ which imports cysteine into the cell, along with glutamate‐cysteine ligase (GCL) that catalyzes the rate‐limiting step in glutathione (GSH) biosynthesis.^26^ Nrf2 regulates glutathione peroxidase (GPx) to maintain peroxides level in order to produce oxidized glutathione (GSSG),^27^ and glutathione reductase‐1 (GSR1), which reduces GSSG to maintain intracellular levels of reduced GSH.^22^ The induction of thioredoxin‐1 (Txn1),^29^ thioredoxin reductases‐1 (TrxR1),^30^ peroxiredoxins (Prxs),^28^ and sulfiredoxin‐1 (Srxn1)^31^ is also subject to Nrf2 regulation for the reduction of oxidized protein thiols and the removal of peroxides. Importantly, NADPH is required as a coenzyme for many xenobiotic metabolism and antioxidant enzymes, including AKR, NAD(P)H: quinone oxidoreductase‐1 (NQO1), and GSR1. Additionally, Nrf2 regulates four NADPH‐generating enzymes, namely glucose‐6‐phosphate dehydrogenase (G6PD), 6‐phosphogluconate dehydrogenase (PGD), isocitrate dehydrogenase‐1 (IDH1), and malic enzyme‐1 (ME1). Heme and quinone both transfer electrons and are therefore direct sources of free radicals and ROS. Moreover, Nrf2 regulates heme oxygenase‐1 (HO‐1) and NQO1 to catalyze heme and quinone degradation.^11^

Phase III drug transporters extrude endogenous xenobiotics and conjugated metabolites out of cells, including multidrug resistance‐associated proteins (MRPs), P‐glycoprotein (P‐pg), and organic anion‐transporting polypeptide (OATP).^37^

### 2.3 General function of Nrf2 pathway

Nrf2 downstream genes, illustrated in Figure 1, are involved in intracellular redox balancing, xenobiotic response, metabolism, and cell survival. Living cells require cellular homeostasis for metabolic process and the ability to rapidly respond to various stresses imposed by toxic exposure. ROS and toxic metabolites generated by ROS‐mediated cell damage give rise to oxidative stress that is apparently adverse to cell survival and further contribute to the induction of tumorigenesis.^47^ The Nrf2‐mediated antioxidant response is one of the major cellular defense mechanisms that protect against oxidative stress. The activation of Nrf2 signaling pathway scavenges ROS and RNS by upregulating the expression of multiple drug‐metabolizing enzymes, such as GCL, AKR, UGT, and MRPs. Likewise, Nrf2‐regulated metabolic pathways including pentose phosphate pathway (PPP) and fatty acid pathway, which are essential contributors to the maintenance of cellular redox and normal cell proliferation.

### 2.4 Nrf2 in cancer prevention

There are abundant evidences that the activation of Nrf2 is able to suppress carcinogenesis, especially in its early stage. Under the physiological condition, Nrf2 maintains the cellular redox homeostasis and exerts anti‐inflammatory functions and further anticancer activities, hence supports cell survival.

### 2.5 Nrf2 maintain cellular redox homeostasis

Under the physiological condition, Nrf2 implements its general function of maintaining cellular redox homeostasis and regulating cell growth, which is molecular basic of Nrf2 to prevent the tumorigenesis.

The physiological relevance between Nrf2, cellular redox homeostasis, and tumorigenesis has been shown by numerous studies. Nrf2‐deficient mice are generally more susceptible to redox disturbances and easily developing drug toxicity.^48^, ^49^ For instance, loss of Nrf2 initiates a detrimental cascade of reduced GST expression and elevates ROS level, ultimately leading to DNA damage and tumorigenesis.^50^ *NFE2L2* gene knockout mice are more sensitive to exogenous chemicals, leading to the formation of bleomycin‐induced pulmonary fibrosis and hyperoxic lung injury^51^ as well as acetylhydrolase‐induced liver cancer.^52^ Oxidative tissue damage after ischemia and reperfusion, including that resulting in noise‐induced hearing loss, is effectively suppressed by Nrf2 activation through its antioxidant function.^53^

### 2.6 Nrf2 exerts an anti‐inflammatory activity

Inflammation induces the generation of ROS and other reactive species, causing DNA damage, activating oncogenes or inactivating tumor suppressor genes, and stimulating proliferation of initiated cells, metastasis, and angiogenesis. Inflammation‐induced carcinogenesis is attributed as part of cytokines that retaining the pro‐inflammatory properties. Nrf2 regulates the expression of antioxidant and cytoprotective enzymes to guard against oxidative electrophilic insults and inhibit excessive production of pro‐inflammatory mediators, thereby constitute to the fundamental line for the chemoprevention of inflammation‐associated cancer. The Nrf2‐regulated Gpx and Trx are verified to suppress inflammatory response.^54^, ^55^ Cyclooxygenase‐2 (COX‐2), inducible nitric oxide synthase (iNOS), and tumor necrosis factor (TNF‐α) are significantly elevated in the Nrf2‐deficient mice, indicating an inhibitory function of Nrf2 toward pro‐inflammatory mediators.^56^ Additionally, the overexpression of HO‐1, a Nrf2 downstream target, attenuates TNF‐α‐induced oxidative stress and interleukin (IL)‐3 via the suppression of the DNA‐binding activity of activator protein‐1 (AP‐1).^57^ Similarly, Nrf2‐dependent induction of NQO1 downregulates lipopolysaccharide (LPS)‐induced expression of TNF‐α and IL‐1β, thereby impairs the inflammatory response.^58^ To conclude, another significant role of Nrf2 is to suppress inflammatory response and protect cells against inflammatory injury and inflammation‐induced carcinogenesis.

### 2.7 Nrf2 inhibits tumorigenesis

The activation of Nrf2/Keap1 pathway is one of the most important mechanisms in anti‐tumorigenesis. In tumor microenvironment, Nrf2 is activated by tumor suppressor genes *BRCA1* and protein p21 via the inhibition of Keap1/Nrf2 complex formation^59^, ^60^ and is blocked by oncogene *Fyn*‐mediated degradation.^61^ The expression of antioxidant and phase II enzymes was found to be abrogated in the Nrf2‐deficient mice. The aggravated oxidative stress caused by quinone rendered the Nrf2‐deficient mice to be more prone to skin cancer, while the expression of NQO1 and GST regulated by Nrf2 decreased when compared to wild‐type mice.^62^ Similarly, Nrf2‐deficient mice treated with carcinogens develop the much larger number of tumors in the forestomach,^63^ liver,^64^ and urinary bladder^65^ compared with that of the wild‐type mice, suggesting that Nrf2 protects against inflammation‐induced carcinogenesis.

Many compounds from plants such as sulforaphane (an isothiocyanates in broccoli), curcumin, carnosol, and resveratrol, certain synthetic chemicals such as oltipraz (an antischistosomal drug and a 1,2‐dithiole‐3‐thione derivative) as well as synthetic oleanane triterpenoids have been discovered to exert chemopreventive activities through the induction of Nrf2/ARE‐regulated genes. Most of these Nrf2 activators affect Nrf2 activity by modifying intermolecular disulfide bonds between two Keap1 molecules at Cys273 and Cys288, to stabilize and enhance nuclear accumulation of Nrf2.^66^ For instance, sulforaphane induces the transcription of phase II enzymes, while inhibits phase I enzymes expression and facilitates cancer cell apoptosis via p53 mechanisms.^67^ Additionally, sulforaphane targets NF‐κB^68^ as well as both *JUN* and *FOS* of the AP‐1 complex to perform an anti‐inflammatory effect.^69^ Another well‐studied chemopreventive drug, synthetic oleanane triterpenoids, inhibits carcinogenesis through the suppression of oncogenes transcription as diverse as *K‐Ras, TP53, Brca1*, and *Erbb2* in many organs such as pancreas, breast, or lung.^70^, ^71^ Indeed, many drugs that function by enhancing the Nrf2 activity are now in clinical trials for numerous indications. These studies show that Nrf2 plays an essential role in tumorigenesis inhibition and can be further strengthened by chemopreventive compounds.

## 3 NRf2 IN CANCER TREATMENT

**Figure 2:**
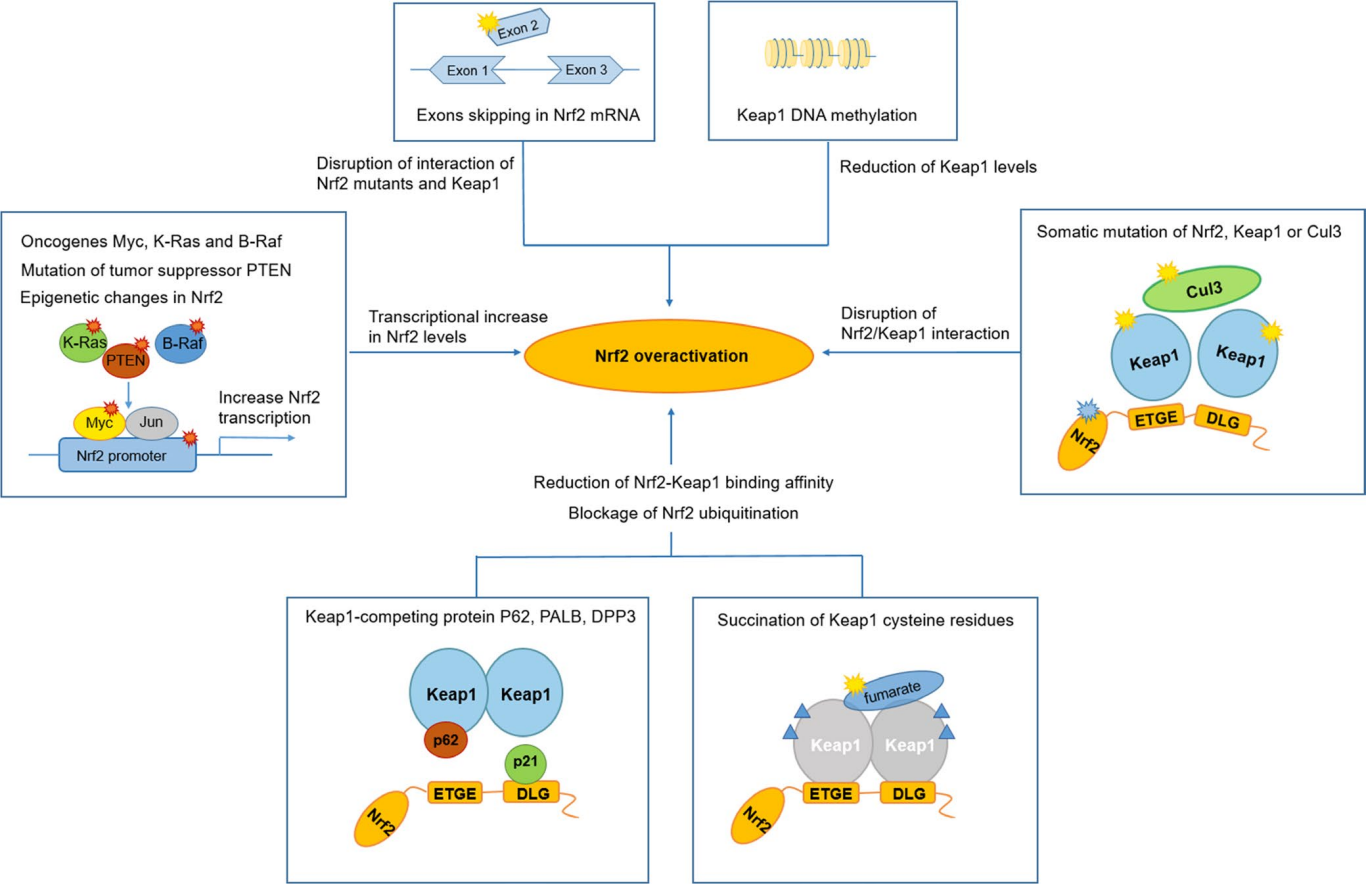
Mechanisms for Nrf2 overactivation in cancers. Oncogenes (*Myc*, *K‐Ras* and *B‐Raf*), mutations of tumor suppressor *PTEN*, and epigenetic changes in Nrf2 lead to the transcriptional increase in Nrf2 levels. Keap1 methylation transcriptionally reduces Keap1 levels. Exon skipping of Nrf2 and somatic mutations of Nrf2, Keap1, or Cul3 disrupt Nrf2/Keap1 interaction. Succination of Keap1 cysteine and Keap1‐competing protein such as p62 results in the reduction of Nrf2‐Keap1‐binding affinity and blockage of Nrf2 ubiquitination

The constitutive activation of Nrf2 promotes the development of various cancers and notably increases the cancer resistance. Clinically, the excessive expression of Nrf2 is always observed with poor prognosis.^72^ Several mechanisms by which Nrf2 signaling pathway is constitutively activated in various cancers have been described (Figure 2): (a) facilitative Nrf2 transcription by oncogenic *Myc*, *K‐Ras*, and *B‐Raf* mutation via mitogen‐activated protein kinases (MAPKs)^47^; (b) somatic mutations in Keap1, Nrf2, or Cul3 disrupting Nrf2/Keap1 interaction^73^, ^74^, ^75^; (c) loss of exons in Nrf2 mRNA resulting in Nrf2 mutants that do not interact with Keap1^76^; (d) epigenetic DNA methylation of Keap1 reducing Keap1 expression level^77^; (e) mutations of tumor suppressor gene *PTEN* and epigenetic changes amplifying Nrf2 level^36^; (f) Keap1‐competing proteins such as partner and localizer of *BRCA2*(PALB2), dipeptidyl peptidase 3 (DPP3), Wilms tumor gene on X chromosome (WTX), p21 and p62 disrupt Nrf2/Keap1 interaction^60^, ^78^, ^79^, ^80^; (g) succination of Keap1 cysteine due to the loss‐of‐function mutations in fumarate hydratase resulting in the reduction of Nrf2‐Keap1‐binding affinity and blockage of Nrf2 ubiquitination.^81^ Cancer cells acquire new characteristics through Nrf2 hyperactivity including infinite cell growth and proliferation, avoidance of apoptosis, enhanced chemoresistance and radioresistance, and induction of angiogenesis and metastasis. The mechanisms of persistent activation of Nrf2 in cancer promotion are discussed in many respects.

### 3.1 Nrf2 promotes cancer cell growth and proliferation

Cancer cells differ from normal cells by its enormous growth and proliferative capacity, which is often observed with Nrf2 overactivation. The reduced state of GSH is indispensable for cell proliferation due to its detoxification, antioxidant defense function, etc. The excessive activation of Nrf2 greatly facilitates transcriptions of several genes involved in the formation of NADPH, the main cofactor in GSH synthesis.^82^ Nrf2 overactivation in cancer cells results in the significantly elevated expression of G6PD, TKT, PGD, and other metabolic enzymes. These highly expressed metabolic enzymes promote glucose and glutamine metabolism in the PPP and augment the synthesis of purine and amino acids, all of which contribute to metabolic reprogramming for cell proliferation.^36^ Nrf2 also regulates genes involved in metabolism of fatty acids and other lipids.^83^ Moreover, microRNA *miR‐1* and *miR‐206* are under control of Nrf2 to direct carbon flux toward PPP and tricarboxylic acid (TCA) cycle.^84^

Nrf2 promotes cell proliferation not only by metabolic reprogramming. The cell cycle regulation is closely related to Nrf2 activity by its target proliferation‐associated genes, including *Bmpr1a, Igf1, Itgb2, Jag1*,* and Pdgf‐c*.^85^ G2/M‐phase arrest is observed with Nrf2 deficiency, suggesting that Nrf2 is necessary for cell cycle regulation by controlling the inhibitory cell‐cycle regulators.^86^ The activation of the phosphoinositide 3‐kinase (PI3K)/protein kinase B (AKT) signaling in hepatocytes was attenuated in Nrf2‐deficient mice,^87^ revealing that persistently elevated Nrf2 promotes the phosphorylation of AKT, GSK3, and the PPP, establishing a crosstalk with the PI3K/AKT pathway to enhance anabolic efficiency.^36^ Besides, Nrf2 participates in healthy mitochondrial maintenance by controlling substrate availability for mitochondrial respiration, inducing mitochondrial biogenesis, and removing damaged mitochondria.^88^ A recent study found that loss of Nrf2 led to impaired mRNA translation in pancreatic cancer cells due to the defects in epidermal growth factor receptor (EGFR) signaling pathway and oxidation of specific translational regulatory proteins, demonstrating that Nrf2 is necessary for cancer cell maintenance by modulating mRNA translation.^89^

### 3.2 Nrf2 suppresses cancer cell apoptosis

In addition to the ability of unlimited proliferation, cancer cells are also characterized to escape from cell apoptosis. Cancer cells frequently express high levels of ROS‐scavenging enzymes, which confer resistance to ROS‐mediated cell death. The excessive activation of Nrf2 counterbalances the accumulated ROS by upregulating antioxidant enzymes, for instance, GCL and GSR that required for the synthesis of GSH.^90^ Nrf2 contributes to the avoidance of cancer cell death also by interacting with other pathways within the cells. The tumor suppressor p53 triggers cell growth arrest and apoptosis partly dependent on decreased Nrf2 activity via counteracting the expression of x‐CT, NQO1, and GST.^91^ Additionally, Nrf2 downstream target glutathione‐S‐transferase pi 1 (GSTP1) suppresses the activation of proapoptotic c‐Jun N‐terminal kinases (JNKs).^92^ Another downstream target p62 creates a positive feedback loop via initiation of selective autophagy of Keap1, to further block apoptosis.^93^ Moreover, Nrf2 mediates the upregulation of antiapoptotic protein B‐cell lymphoma 2 (Bcl‐2) which leads to the decrease in etoposide‐mediated cell apoptosis and increases cell survival.^94^ TNF‐induced cell death is also suppressed through the upregulation of HO‐1.^95^ In conclusion, the continuous activation of Nrf2 in cancer cells inhibits cell apoptosis, enhances the survival benefits of oxidized damaged cells, thereby promoting cancer development and progression.

### 3.3 Nrf2 promotes self‐renewal of cancer stem cells

Cancer steam cells (CSCs), characterized by their self‐renewal and differentiation properties, are thought to be partly responsible for anticancer drug resistance and tumor relapse after therapy. The self‐renewal capacity of CSCs is largely due to elevated expression of antioxidant enzymes, drug transporters, cell cycle quiescence, and enhanced DNA repair capacity.^96^ Nrf2 expression has been proved to regulate cell cycle quiescence and cell fate determination as well as reconstitution capacity of hematopoietic stem cells (HSCs).^97^, ^98^ These findings are consistent with the results in Drosophila intestinal stem cells; the cell quiescence was obtained through the reduction in ROS level due to Nrf2 upregulation.^99^ The persistent activation of Nrf2 also drives the myeloid differentiation and represses erythroid and lymphoid differentiation.^97^ Numerous studies have demonstrated that Nrf2 plays a vital role in various CSCs survival and self‐renewal so far. Nrf2 level was higher in glioma stem cells (GSCs) than non‐GSCs fraction,^100^ and Nrf2 overactivation induced its transcriptional network to perform self‐renewal capacity in GSCs with the treatment of anticancer drugs.^101^ Similarly, in neural stem cells (NSCs), amyloid‐β (Aβ1)‐induced toxicity was alleviated by Nrf2 overexpression along with increased expression of GCL, NQO1, and HO‐1, while Nrf2 deficiency enhanced the Aβ1‐42‐induced reduction of neuronal differentiation.^102^ In mesenchymal stem cells (MSCs), the continuous activation of Nrf2 reduced oxidative stress‐induced apoptosis and cytotoxicity.^103^

### 3.4 Nrf2 promotes anti‐inflammation activities

The activation of Nrf2/ARE signaling pathway plays a critical role in alleviation of chronic inflammation, which is associated with cancers. Since Nrf2 positively regulates a large number of cytoprotective proteins, elimination of ROS has been widely accepted as the molecular basis of Nrf2‐mediated anti‐inflammation. This has been proved by several reports that Nrf2‐deficient mice showed greater LPS‐induced pulmonary inflammation, which could be attenuated by Nrf2 inducers.^104^, ^105^ Nrf2 represses the activation of pro‐inflammatory genes and potentiates the anti‐inflammatory signaling. Nrf2 interferes with LPS‐induced transcriptional upregulation of pro‐inflammatory cytokines in macrophages (IL‐6 and IL‐1β), by binding to proximity of corresponding genes and inhibiting RNA polymerase II recruitment.^109^ Similarly, Nrf2 upregulates pro‐inflammatory chemokine IL‐8 to promote anti‐inflammation activities. Nrf2 activation is directly associated with the induction of IL‐8 expression by increasing the half‐life of IL‐8 mRNA.^110^ Indirectly, hypoxia‐inducible factor (HIF‐1) induction attenuated Nrf2‐dependent IL‐8 expression in human endothelial cells, suggesting a crosstalk between Nrf2 and HIF‐1 to promote IL‐8 expression.^111^ Likewise, Nrf2 activation protects cells against sepsis and H_2_O_2_‐mediated injury via p38/MAPK pathway to modulate pro‐inflammatory cytokine including monocyte chemotactic protein 1 (MCP1), vascular cell adhesion molecule 1 (VCAM1), TNF, and macrophage inflammatory protein 2 (MIP2).^112^, ^113^ The recent studies revealed that Nrf2 crosstalks with other important inflammatory pathway to bring coordinated innate immune response in form of inflammation. The toll‐like receptors (TLRs) signaling pathway plays a key role in modulating immune responses by eliciting inflammatory reactions via the production of inflammatory cytokines (TNF‐α, IL‐6), chemokines (IL‐8, MIP2), and interferons (type‐I). Several cell‐specific kinases (protein kinase C, MAPK, Bruton's tyrosine kinase, and PI3Ks) are known to mediate crosstalk between TLR and Nrf2 either by regulation of p62‐mediated autophagy,^93^ expression of anti‐inflammatory proteins (eg, HO‐1, NQO1, SOD),^114^, ^115^ or suppression of pro‐inflammatory cytokines (eg, IL‐6, IL‐1β).^117^ Nrf2 inducers attenuate TLR‐driven inflammation as well as pro‐inflammatory cytokines (IL‐6, IL‐1β, TNF) in sepsis or inflammatory disorders, while TLRs agonists may also act as activator of Nrf2 pathway, augment the expression of antioxidant proteins, and contribute to cell survival.^108^, ^114^ Reportedly, Nrf2 crosstalks with TLR also result from the interactions of former with nuclear factor kappa‐B (NF‐κB) pathway. Nrf2 target gene *HMOX1* possesses prominent anti‐inflammatory despite of its cytoprotection role. Nrf2 activation inhibits NF‐ĸB pathway mainly by elevating HO‐1 expression and antioxidant defenses which neutralize ROS and detoxifying chemicals.^118^ It also controls NF‐κB pathway through Keap1, which stabilizes the NF‐ĸB inhibitor (IKB)‐α and represses degradation of NF‐ĸB kinase inhibitor (IKK)‐β, leading negatively to the regulation of NF‐κB pathway.^119^ NF‐ĸB exerts its negative effect on Nrf2‐driven gene expression through p65, along with MafK and histone deacetylase3 (HDAC3). Moreover, NF‐ĸB‐mediated transcription reduces Nrf2 activation by competing with Nrf2 for CBP.^120^ Since Nrf2 contributes to anti‐inflammatory effects and cell survival, Nrf2 inducers may be further explored for novel therapeutic treatment in inflammatory diseases and inflammation‐associated cancers.

### 3.5 Nrf2 promotes angiogenesis

The induction of angiogenesis is one of the hallmarks of inflammation‐induced carcinogenesis. Angiogenesis is under control in normal physiological processes, whereas it is continuously activated in cancers. HO‐1 overexpression augmented vascular endothelial growth factor (VEGF) production and VEGF‐mediated angiogenic activities by increasing proliferation, migration, the formation of tubes on Matrigel, and the outgrowth of capillaries from endothelial spheroids.^121^ The proangiogenic role of VEGF/Nrf2‐dependent pathway induced by Nrf2 activation was proved in rat gastric epithelial cells^122^ as well as glioma cells^123^ and pancreatic cancer cells.^124^ Nrf2 blockade inhibited hypoxia‐induced activation of HIF‐1α/VEGF signaling to suppress tumor angiogenesis, indicating a crosstalk mechanism between Nrf2 and HIF‐1α in angiogenesis.^125^

### 3.6 Nrf2 enhances chemoresistance of cancer cells

Multiple factors contribute to the buildup of the chemoresistance of cancer cells. Several mechanisms of chemoresistance have been proposed to be associated with the pharmacokinetics and pharmacodynamics. Chemical activation of Nrf2 by pretreatment with tBHQ increases the survival of neuroblastoma cell in response to three chemotherapeutic drugs, namely cisplatin, doxorubicin, and etoposide.^12^ Drug resistance toward tamoxifen in MCF‐7 cell lines driven by Nrf2 activation is attributed to antioxidant enzymes, including Trx, Prx, and GCL.^11^ Several phase II enzymes regulated by Nrf2 have also proved to be chemoresistance. The downregulation of Nrf2 by siRNA rendered cancer cells more susceptible to cisplatin due to the suppression of NQO1.^126^ The transient transfection of GSTP1 enhances the drug resistance to adriamycin, cisplatin, melphalan, and etoposide in colon cancer cells.^127^ The chemoresistance role of Nrf2 is consistent by repressing HO‐1 in lung carcinoma A549 cells.^32^ Notably, the depletion of GSH elevated the drug sensitivity to cisplatin, and the amplification of cellular GSH level protects against toxic effects of cytotoxic drug both in vivo and in vitro, indicating a role for GSH in chemoresistance.^126^, ^128^ Moreover, Nrf2 overactivation drives the expression of MRPs, thus reduces anticancer drug accumulation in cancer cells.^129^, ^130^ These efflux transport proteins have been an attractive target for many researchers to prevent chemoresistance, such as identifying peptides that hindering MRPs, finding the way to downregulate MRP genes, or designing new drugs that are not substrate of MRPs. Peroxisome proliferators activator receptors gamma (PPARγ) has been reported to effectively suppress cancer progression.^132^ When treated with Keap1 shRNA, PPARγ of the non‐small cell lung cancer (NSCLC) cells increase, and cells were more susceptible to arsenic trioxide, etoposide, and doxorubicin, suggesting that Nrf2 contributes to drug resistance by the downregulation of PPARγ.^133^ To summarize, when Nrf2 is overactivated in cancer cells, specific antioxidant enzymes, phase II enzymes, and transporters are excessively expressed. These downstream proteins hinder the entry of drug into the cells and block the drugs decomposition rate and efflux in cells to decrease the efficacy of drugs.

### 3.7 Nrf2 enhances radioresistance of cancer cells

Radiation therapy leads to cancer cell death through the generation of ROS with the combination of chemotherapeutic agents.^134^ However, persistent activation of Nrf2 significantly enhances the cancer cell resistance to ROS by upregulating antioxidant enzymes and attenuating the sensitivity to cytotoxic chemotherapeutic agents.^135^ The upregulated Nrf2 in radiation therapy is found to be associated with higher expression of HO‐1, NQO1, Prx, and other Nrf2 downstream targets that promote GSH synthesis. Nrf2 also establishes the crosstalk with other radioresistance‐related signaling pathways such as HIF‐1^136^ and NF‐kB^137^ against radiotherapy. Reduction of Nrf2 levels in NSCLC cells led to a dramatic increase in endogenous ROS levels. Similarly, significantly higher γ‐irradiation‐induced protein carbonyl levels were observed in Nrf2‐depleted lung cancer cells. These findings conclude that Nrf2 confers to radioresistance against ionizing radiation toxicity. Targeting Nrf2 activity in tumors may be an effective method to avoid radioresistance.^138^

### 3.8 Nrf2 inhibitors

As the pro‐tumorigenic role of Nrf2 in cancer cells has been clearly shown, pharmacological modulation of the Nrf2 pathway offers novel therapeutic opportunities for oxidative stress‐related diseases, such as cancer, diabetes, Alzheimer's disease, arteriosclerosis, inflammation, and myocarditis. Most of currently known Nrf2 activators (eg, curcumin, sulforaphane, and oltipraz) lack specificity, leading to the rising risk of “off‐target” toxic effects due to their ability to react with the cysteine residues of other enzymes and proteins. Another problem is the metabolic instability, poor membrane permeability, and low bioavailability of some Nrf2 modulators, such as curcumin and its analogs. Demanding the modulators of Nrf2‐Keap1 pathway, therefore, requires not only potent efficacy but also good bioavailability and specificity.

A number of natural compounds and synthetics have been identified as Nrf2 inhibitors, but currently none has yielded strong and practicable results. Ascorbic acid (Vitamin C) prevents Nrf2‐mediated redox imbalance caused by hydrogen peroxide and UV irradiation, however, cannot efficiently protect cells from apoptosis by metabolic dysregulation.^139^ Retinoic acid is a metabolite of dietary vitamin A, which binds to RARα and inhibits Nrf2 binding to the ARE.^140^ These vitamins and their derivatives do not exert potent inhibitory of Nrf2, and the high concentrations may result in opposite effects.

A large number of substances extracted from natural compounds were proved to exert Nrf2 inhibitory effect. EGCG (Epigallocatechin 3‐gallate), the major polyphenol found in green tea, shows the capability of suppressing Nrf2 activity and reducing HO‐1 expression, although the concentrations of EGCG required for its inhibitory effect were high (>200 μmol/L).^141^ Luteolin, a polyphenolic flavonoid, elicited a dramatic reduction in Nrf2 at both the mRNA and the protein levels in A549 cells^142^ and cholangiocarcinoma cells,^143^ resulting in downregulation of Nrf2 target genes. However, Luteolin shows an opposite effect of activating Nrf2 in several other cell lines (eg, PC12, HepG2)^144^, ^145^ and rat models (eg, colorectal cancer).^146^ Hence, luteolin function as an Nrf2 modulator still needs to be considered. Other flavonoids activate Nrf2 pathway includes procyanidin,^147^ apigenin,^148^ and chrysin.^149^ Overall, the effects of flavonoids on the Nrf2/ARE pathway appear to be cell type‐specific, concentration dependent, and stage dependent and may vary depending on cancer properties.

Brusatol, a component of *Brucea javanica* seeds, strongly reduces the protein level of Nrf2 in A549 cells, sensitizing them to cisplatin and other chemotherapeutic drugs.^150^ Nevertheless, Brusatol was proved lately to be a nonspecific inhibitor as it rapidly and significantly reduces the level of a large amount of proteins, demonstrating its potential role as a protein translation machinery inhibitor.^151^ Ochratoxin A (OTA), produced by *Aspergillus*and *Penicillium*species, is both a nephrotoxin and a renal carcinogen. OTA dramatically alleviates the mRNA level of Nrf2 as well as GCL and GST levels in LLC‐PK1 cells.^152^ There are several potential mechanisms for OTA‐induced Nrf2 inhibition: (a) blocking nuclear import of Nrf2; (b) reducing Nrf2‐DNA binding; (c) epigenetic modification of Nrf2 through upregulation of *miR‐132*.^153^ In another study, reduction by OTA in Nrf2‐dependent gene expression was observed in the kidney, but not liver.^154^ Coupled with the facts that OTA possess severe toxicity, this may not be an ideal agent for clinical purpose.

A few substances isolated from traditional herbs are also identified to be Nrf2 inhibitors. Cryptotanshinone, extracted from *Salvia miltiorrhiza*, inhibits Nrf2 protein expression and sensitizes A549 cells to cisplatin. However, its poor bioactivity is a major obstacle to utilize cryptotanshinone in clinical therapy.^155^ Wogonin (5,7‐dihydroxy‐8‐methoxyflavone), isolated form *Scutellariae radix*, is known to have anti‐inflammatory, antiviral, and anticancer effects. It reduces Nrf2 activity by suppressing the PI3K/Akt and Stat3/NF‐κB signaling and reverses chemoresistance.^156^, ^157^ Inversely, wogonin has also been reported to activate Nrf2 expression to exerts its antioxidant and anti‐inflammatory effects. The roles of wogonin in Nrf2 modulatory are contradictory though wogonin has low toxicity and good pharmacokinetic properties.^157^

Some approved medicines for the treatment of various diseases are found to block Nrf2 activity. *All‐trans*‐retinoic acid (ATRA), approved for medical use for the treatment of acne and acute promyelocytic leukemia, has been proposed as a specific Nrf2 inhibitor, which enables Nrf2 forms a complex with retinoid X receptor alpha (RARα), blocking activation of the Nrf2 pathway to suppress chemoresistance.^140^ ATRA at different concentrations elicits distinct Nrf2 modulation. Micromolar concentration of ATRA suppresses Nrf2 activation; in contrast, highly toxic concentration (10^−7^‐10^−5^ mol/L) of ATRA activates Nrf2 and induces Nrf2 target genes.^158^ Halofuginone is the derivative of *febrifugine* which is a bioactive component of the traditional Chinese medical herb *Dichroa febrifuga*. It has now been tested in phase II clinical trials for cancer and fibrotic diseases.^159^, ^160^ Recently, halofuginone was found to decrease Nrf2 protein synthesis by inhibiting prolyl‐tRNA synthetase, although halofuginone is not a specific inhibitor of Nrf2. However, cotreatment with halofuginone strengths the effects of conventional anticancer drugs in a xenograft tumor model, such as cisplatin or doxorubicin, illustrating a novel therapeutic cotreatment that alleviates Nrf2‐mediated chemoresistance.^161^ Metformin, a drug widely used in the treatment of type 2 diabetes, reduces mRNA and protein levels of Nrf2 through the suppression of Raf/ERK/Nrf2 signaling.^162^ Another postulated mechanism is that metformin may induce *miR‐34a* that decreases the protein expression of Nrf2 through the Sirt1/PGC‐1α/Nrf2 signaling.^163^ Conversely, metformin represses cancer cell growth and induces autophagy through the AMPK/mTOR pathway, which could also potentially activates Nrf2 in a p62‐dependent manner.^164^ Another therapeutic drug for type II diabetes, trigonelline inhibits Nrf2‐dependent proteasomal activity and interferes with Nrf2 nuclear import at low concentrations (0.0001‐1 μmol/L).^165^ The research of metformin and trigonelline in combination therapy with cancer chemotherapeutic drugs is currently ongoing. Clobetasol propionate, a glucocorticoid for various skin disorders, has been demonstrated as a potent Nrf2 inhibitor due to its capability of preventing nuclear accumulation and promoted β‐TrCP‐dependent degradation of Nrf2 in a glucocorticoid receptor‐ and a GSK3‐dependent manner. The combination of clobetasol propionate and rapamycin is proposed as a potential therapeutic strategy for tumors harboring both Keap1 and LKB1 mutations. On the basis of screening, the study also proposed that three classes of drugs: glucocorticoids, cardiac glycosides, and antimetabolites have the potential to be potent Nrf2 inhibitors.^166^

Some studies have discovered small molecule inhibitors of Nrf2 by high‐throughput screening of compound libraries. ML385, a thiazole‐indoline compound that specifically binds to Neh1 domain of Nrf2 and interferes with the binding of the MafG‐Nrf2 interaction to inhibit Nrf2 pathway. In preclinical models of Nrf2‐mediated NSCLC, ML385 shows significant antitumor activity in combination with carboplatin.^167^ ARE expression modulator 1 (AEM1) contains a thienopyrimidine structure and inhibits mRNA and protein expression of Nrf2. Interestingly, AEM1 exerts its inhibitory role by an unknown mechanism other than altering the protein levels of Nrf2 or Keap1. A cyclin‐dependent kinases (CDK) inhibitor, PHA‐767491, was identified to be a potent inhibitor of Nrf2 transcriptional activity through a quantitative high‐throughput screening.^168^ The mechanism of its inhibitory effect needs further investigation. IM3829 (4‐[2‐Cyclohexylethoxy] aniline) decreases Nrf2 mRNA and protein levels, and combined treatment with radiation is able to significantly inhibit cancer cell survival, suggesting a promising radiosensitizer in lung cancer treatment.^135^ By screening a siRNA library that targets the majority of the druggable genome, a study found that Grassypeptolide A, an active peptide isolated from marine cyanobacteria contains actin, is able to attenuate Nrf2 activity.^169^ However, the cytotoxicity and bioactivity of these compounds are still unclear.

Moreover, miRNAs that negatively regulate Nrf2 expression offer additional putative targets to manipulate Nrf2 pathway. *miR‐144* was the first identified miRNA for the decreasing of Nrf2 protein level via targeting two distinct sites in the Nrf2 untranslated region.^170^ Similarly, *miR‐28* targets the 3'UTR of Nrf2 mRNA and decreases Nrf2 protein expression.^171^

Identified proteins that involved in the regulation of Nrf2 signaling pathway, such as protein kinases, provide more opportunities to modulate Nrf2 pathway. For instance, GSK‐3β has been observed to indirectly modulate Nrf2 via tyrosine‐protein kinase Fyn phosphorylation. Fyn translocates to the nucleus resultant to GSK‐3β phosphorylation, and in turn phosphorylates Nrf2, which stimulates its activation. GSK‐3β inhibitors for the blockage of Nrf2 have been studied for neurodegenerative diseases including Alzheimer's disease (AD) and brain ischemia.^172^ This allows pre‐existing drug, such as kinase inhibitors, to inhibit Nrf2 activation and potentially represses cancer development.

## 4 CONCLUDING REMARKS AND FUTURE PERSPECTIVES

Overall, the role of Nrf2 activation in cancer is paradoxical and requires further exploration. For the prevention of chronic diseases and cancer in which oxidative and inflammatory stress contributes to the pathogenesis, enhancing Nrf2 activity is still a traditional and effective approach. However, studies in the past few decades have proposed that the overactivation of Nrf2 promotes cancer cell growth and proliferation, blocks cell apoptosis, strengthens CSCs self‐renewal capacity, most importantly, enhances the chemoresistance and radioresistance of cancer cells. Hence, it is reasonable to consider that blocking the Nrf2 activity in fully malignant cells may be a considerable way for cancer prevention. Previous works have provided a large number of Nrf2 inhibitors that regulate Nrf2 at different levels and proved the anticancer effect of Nrf2 inhibition. However, currently, none has yielded strong and practicable results. A few small molecules discovered currently display promising availabilities in Nrf2 inhibition, which still needs to be further investigated and optimized. An ideal inhibitor for clinical application requires not only potent efficiency and specificity but also less toxicity, good bioactivity, and pharmacokinetics. A better strategy is probably not only to focus on directly targeting Nrf2 but also to explore indirect methods such as the inhibition of upstream miRNAs or protein kinases.

## CONFLICT OF INTEREST

There are no conflicts of interest to this work.

## AUTHOR CONTRIBUTIONS

Shijia Wu wrote the original draft. Hong Lu and Yongheng Bai reviewed and edited the manuscript.
